# Safety and efficacy of flexible articulated instrument (ArtiSential^®^) in laparoscopic surgery for rectal cancer

**DOI:** 10.1186/s12893-025-02841-9

**Published:** 2025-05-02

**Authors:** Jong-Sung Ahn, Jesung Park, Seung-Bum Ryoo, Min-Jung Kim, Ji-Won Park, Seung-Yong Jeong, Kyu-Joo Park

**Affiliations:** 1 Department of Surgery, Seoul National University Hospital, Seoul National University College of Medicine, Seoul, Korea; 2 Colorectal Cancer Center, Seoul National University Cancer Hospital, Seoul, Korea; 3 Cancer Research Institute, Seoul National University, Seoul, Korea; 4 Division of Colorectal Surgery, Department of Surgery, Seoul National University Hospital, Seoul National University College of Medicine, 101 Daehak-ro (28 Yongon-dong), Jongro‐gu, Seoul, 03080 Korea

**Keywords:** Articulated instrument, ArtiSential, Laparoscopy, Rectal cancer

## Abstract

**Background:**

Laparoscopic surgery for rectal cancer remains challenging because of limited joint motion during dissection in the deep and narrow pelvis. Handheld multiarticulated instruments have been developed to address these limitations. This study aimed to assess the safety and efficacy of a flexible articulated instrument, the ArtiSential^®^ (Livsmed Co, Korea), at reducing the duration of laparoscopic rectal cancer surgery.

**Study design:**

We retrospectively reviewed patients who underwent laparoscopic low or ultralow anterior resection for primary mid to low rectal cancer (tumor distance from anal verge, ≤ 10 cm) performed by a single surgeon in 2012–2022. Patients were divided into groups based on the use of ArtiSential^®^ or straight device, and their clinical characteristics, surgical procedures, pathological findings, postoperative complications, and survival outcomes were analyzed.

**Results:**

The study included 93 patients (articulating group, 32; straight group, 61). Low anterior resection was predominant in both groups, while operative time was significantly shorter in the articulating group (148.08 ± 49.72 vs. 188.13 ± 57.86; *p* = 0.003). Total mesorectal excision quality and resection margin status did not differ between groups. Postoperative complications, including anastomotic leakage, length of hospital stay, 3-year recurrence-free survival rate (90.6% vs. 88.5%, *p* = 0.760), and overall survival rate (100% vs. 85.2%, *p* = 0.092), did not differ between groups.

**Conclusion:**

Use of the flexible articulated instrument (ArtiSential^®^) can reduce operative time without impairing surgical quality or oncologic outcomes. These results suggest that laparoscopic rectal cancer surgery can be performed safely and effectively using a flexible articulated instrument.

**Clinical trial number:**

Not applicable.

**Supplementary Information:**

The online version contains supplementary material available at 10.1186/s12893-025-02841-9.

## Introduction

Colorectal cancer, the third most common cancer worldwide, has an incidence of 19.3/100,000 in 2020 and is the second leading cause of cancer-related death [1]. The incidence of colorectal cancer in South Korea has been rising, reaching 54.3 per 100,000 people, making it the second most prevalent region globally [2]. Rectal cancer accounts for approximately 30–40% of all cancers, with an incidence of 7.3 per 100,000 worldwide (2020) [3]. Sphincter-preserving surgery, such as low or ultralow anterior resection, is the standard treatment for rectal cancer. Owing to advancements in surgical techniques, oncological and survival outcomes have improved, even in cases in which the tumor location is very low and close to the anus.

Laparoscopic surgery for colon cancer has been proven safe and feasible through randomized clinical trials in many countries, with the advantage of early recovery after surgery [4–8]. The Comparison of Open versus laparoscopic surgery for mid and low rectal cancer After the Neoadjuvant chemoradiotherapy (COREAN) trial also verified that laparoscopic rectal cancer surgery is safe and has short-term benefits with no difference in long-term survival outcomes [9–11]. However, subsequent trials could not establish the non-inferiority of successful resection with oncologic safety to laparoscopic rectal cancer surgery, nor did the surgery present a relatively high conversion rate [12–15]. This is because the laparoscopic procedure for rectal cancer has a stiff learning curve, making total mesorectal excision (TME) difficult to perform in cases of a deep narrow pelvis or obesity. Straight instruments such as those used in laparoscopic surgery cannot always reach the pelvic floor, and proper dissection through the exact plane for the TME cannot be achieved [16]. If TME cannot be performed safely, open surgery should be used because it is a long-standing and time-saving procedure. Robotic surgery or transanal rectal resection has been developed to overcome the limitations of laparoscopic surgery; however, its advantages have not yet been demonstrated [17, 18].

A recently developed laparoscopic flexible articulated instrument (ArtiSential^®^, LIVSMED Co., Seongnam, Korea) can be moved relatively freely, even in the deep and narrow pelvis. Moreover, TME can be performed comfortably with reduced operative time. This study aimed to analyze the safety and efficacy of flexible articulated instruments in laparoscopic surgery for rectal cancer.

## Methods

**Fig. 1:**
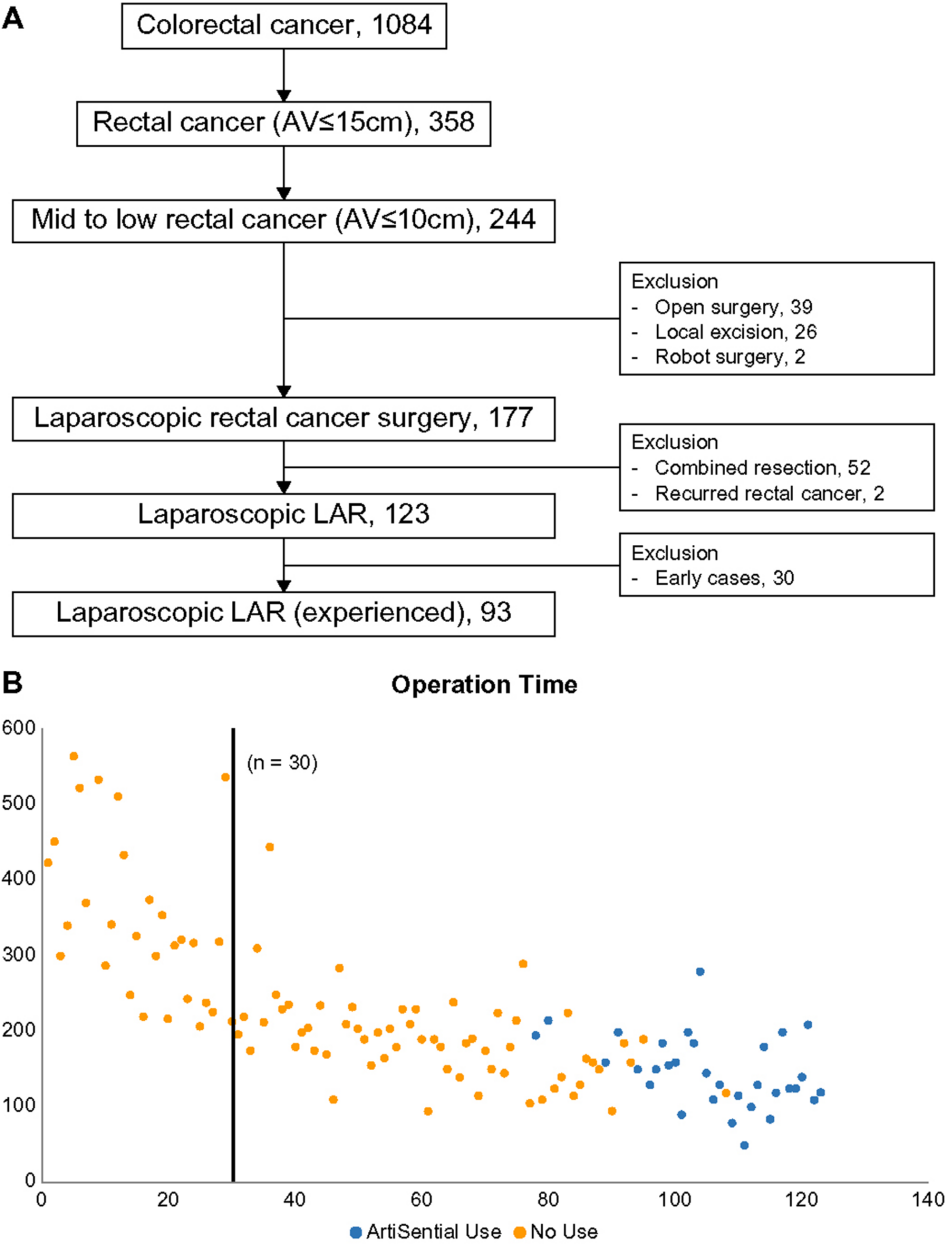
Study profile. (**A**) Flow diagram of patient enrollment process (**B**) Operative time for laparoscopic rectal cancer surgery

We retrospectively analyzed the medical records of consecutive patients who underwent laparoscopic resection for rectal cancer performed by a single experienced surgeon between 2012 and 2022. Among the 358 patients who underwent rectal cancer surgery, 244 had mid to low rectal cancer located within 10 cm of the anal verge; of them, laparoscopic surgery was performed in 177. Patients who underwent combined resection of other organs and surgery for recurrent rectal cancer were excluded. Furthermore, we excluded the first 30 patients to account for the learning curve of laparoscopic low anterior resection. Finally, 93 patients were included in the study (Fig. 1).

**Fig. 2:**
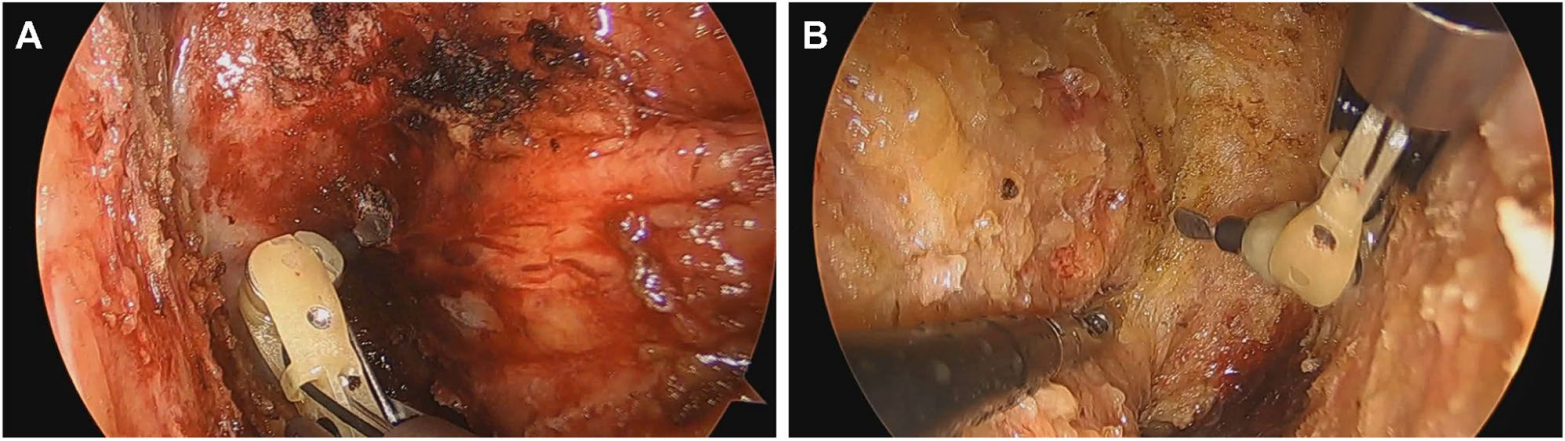
ArtiSential^®^ used during total mesorectal excision at the lowest portion of the pelvic floor. (**A**) Left side (**B**) Right side

The patients were divided into two groups based on the use of ArtiSential^®^ instrument (“articulating group”) or its non-use (“straight group. The “straight group” used a conventional device: Endopath^®^ Electrosurgery PROBE PLUS^®^ II System (EPS02/EPH02; Ethicon Co., OH, US). All operations prior to late 2020 were conducted using a straight device, while those afterward predominantly utilized the ArtiSential. There is, however, a slight overlap where both devices were used during the initial phase of ArtiSential’s adoption. The Endopath^®^ system is a standard electrosurgical device commonly used in laparoscopic surgery with a straight, rigid structure that allows for basic tissue manipulation and dissection [19]. In contrast, the ArtiSential^®^ instrument incorporates a multi-jointed design that mimics the natural movements of the human wrist, providing a 360° range of motion [20]. This flexibility enables surgeons to perform complex maneuvers in confined spaces, such as the deep pelvis, where rigid instruments can be limiting. This design theoretically offers several advantages, including enhanced precision, greater dexterity, and improved access to difficult anatomical areas. These features make the ArtiSential^®^ instrument particularly advantageous for procedures like total mesorectal excision (TME) in rectal cancer. Beginning in 2020, the ArtiSential^®^ was utilized during TME procedures in combination with the EPS02/EPH02 at the lowest portion of the pelvic floor (Fig. 2; Supplement 1). The trocar placement for all rectal cancer surgeries were as follows: A 12 mm camera port would be inserted under the umbilicus. The patient would be in a lithotomy position, and the operator stood on the patients right side, and the first assist on the left side. Both the operator and the assistant each had two working ports at their disposal. The operators main working port that is used by his right hand (patient’s right-lateral quadrant area), is specifically located at the 1/3rd medial side of the straight line between the umbilicus and the right ASIS; this is where the Endopath^®^ or ArtiSential^®^ was used. This location has some advantages of wide range of motion in the narrow pelvis. The trocar placements did not differ between the two groups. In cases where patients underwent neoadjuvant chemotherapy (nCRT), a restaging CT or colonoscopy would typically be performed before surgery. All nCRT patients had diverting stomas created.

A comparative analysis was performed to examine the patients’ clinical characteristics, operative procedures and durations, intraoperative complications, operative histopathology, postoperative complications, and length of hospital stay. Oncological outcomes were also analyzed using a comparative analysis of recurrence and survival outcomes through long-term follow-up.

We performed propensity score matching (PSM) using baseline patient characteristics only. As our primary objective was to evaluate the effect of ArtiSential^®^ usage on surgical outcomes, we excluded operative and pathologic variables from the matching process. Specifically, operation time, estimated blood loss, harvested lymph nodes, intraoperative transfusion, and conversion were not used, as they are direct measures of surgical performance that we intended to study. Likewise, indicators of procedural quality, such as circumferential resection margin (CRM), distal resection margin (DRM), and having ≥ 12 harvested lymph nodes, were omitted to avoid diluting the potential impact of the device. Lastly, tumor histology was excluded because it generally does not affect laparoscopic technical performance or short-term surgical outcomes, and there was no statistical difference in histology between the two groups.

This study was approved by our local institutional review board (H-2208-007-1346), which waived the requirement for written informed consent because of the study’s retrospective nature.

The statistical analyses were performed using SPSS software (version 27.0; IBM Inc., Armonk, NY, US). Categorical variables were compared using Pearson’s χ2 test or Fisher’s exact test, while continuous variables were compared using Student’s t-test or the Wilcoxon rank sum test. The survival analysis was conducted using the Kaplan–Meier method and the log-rank test. Continuous variables are presented as means ± standard deviations, while categorical variables are expressed as percentages. Values of *p* < 0.05 were considered statistically significant.

## Results

### Patients’ characteristics

Among the 93 patients, the flexible articulated instrument (ArtiSential^®^) was used in 32 (34.4%). The mean overall patient age was 61.5 ± 10.8 years (range, 37 − 89 years), and 61 patients (65.6%) were male. The mean body mass index was 24.0 ± 3.4 kg/m^2^ (range, 16.6–40.0 kg/m^2^). The median tumor distance from the anal verge was 8 cm (range, 0–10 cm). No statistically significant intergroup differences were observed in the patients’ clinical characteristics except for poor clinical N category (84.4 vs. 54.1%, *p* = 0.004) and an increased requirement for nCRT (68.8% vs. 34.4%, *p* = 0.002) in the group using the flexible articulated instrument, although after PSM both of these categories were no longer statistically significant (Table 1).

**Table 1:** Patients’ clinical characteristics (*N* = 93)

	Before PSM			After PSM		
	ArtiSential^®^ (*n* = 32)	Straight (*n* = 61)	P value	ArtiSential^®^ (*n* = 32)	Straight (*n* = 20)	P value
Age (years)			0.183			0.229
≥ 65	15 (46.9)	20 (32.8)		15 (46.9)	6 (30.0)	
< 65	17 (53.1)	41 (67.2)		17 (53.1)	14 (70.0)	
Sex			0.167			0.156
M	24 (75.0)	37 (60.7)		24 (75.0)	11 (55.0)	
F	8 (25.0)	24 (39.3)		8 (25.0)	9 (45.0)	
BMI (kg/m^2^)			0.491			0.786
≥ 25	10 (31.3)	15 (24.6)		10 (31.3)	7 (35.0)	
< 25	22 (68.8)	46 (75.4)		22 (68.8)	13 (65.0)	
Comorbidity			0.061			0.914
Yes	26 (81.3)	38 (62.3)		26 (81.3)	16 (80.0)	
No	6 (18.8)	23 (37.7)		6 (18.8)	4 (20.0)	
Preoperative CEA (ng/mL) (*n* = 86)			0.711			0.51
> 5	9 (28.1)	15 (24.6)		9 (28.1)	4 (20.0)	
≤ 5	23 (71.9)	46 (75.4)		23 (71.9)	16 (80.0)	
Distance from AV (cm)			0.160			0.556
≥ 5	27 (84.4)	57 (93.4)		27 (84.4)	18 (90.0)	
< 5	5 (15.6)	4 (6.6)		5 (15.6)	2 (10.0)	
Clinical T category			0.605			0.422
cT0/1	2 (6.3)	9 (14.8)		2 (6.3)	1 (5.0)	
cT2	4 (12.5)	9 (14.8)		4 (12.5)	4 (20.0)	
cT3	21 (65.6)	33 (54.1)		21 (65.6)	14 (70.0)	
cT4	5 (15.6)	10 (16.4)		5 (15.6)	1 (5.0)	
Clinical N category			*0.004			0.7
cN0	5 (15.6)	28 (45.9)		5 (15.6)	4 (20.0)	
cN+	27 (84.4)	33 (54.1)		27 (84.4)	16 (80.0)	
Clinical M category			0.847			0.548
cM0	29 (90.6)	56 (91.8)		29 (90.6)	19 (95.0)	
cM1	3 (9.4)	5 (8.2)		3 (9.4)	1 (5.0)	
Neoadjuvant CRT			*0.002			0.337
Yes	22 (68.8)	21 (34.4)		22 (68.8)	11 (55.0)	
No	10 (31.3)	40 (65.6)		10 (31.3)	9 (45.0)	

AV, anal verge; BMI, body mass index; CEA, carcinoembryonic antigen; CRT, chemoradiotherapy; PSM, propensity score matching

*p < 0.05. Values are shown as n (%)

### Operative outcomes and histopathology results

A low anterior resection was performed in 25 (78.1%) and 56 (91.8%) patients in the use and straight groups, respectively. Diverting stoma formation was encountered significantly more frequently in the articulating group (68.8 vs. 39.3%, *p* = 0.032) because of the higher requirement for nCRT, although after PSM this was no longer statistically significant. Estimated blood loss and intraoperative transfusion requirements did not differ between groups, whereas operative time was significantly shorter in the articulating group (147.2 ± 46.7 min vs. 188.1 ± 57.9 min, *p* = 0.001). After PSM, operative time was still significantly shorter in the articulating group (147.2 ± 46.7 min vs. 193.6 ± 76.1 min, *p* = 0.021). TME quality was high in every case except one (3.1%) in the articulating group and one (1.6%) in the straight group; the difference was not statistically significant. No significant differences were observed in the distances between the circumferential and distal resection margins. Moreover, the American Joint Committee on Cancer stages did not differ between the two groups (Table 2).

**Table 2:** **a** Surgical characteristics (*N* = 93). **b** Surgical histopathology (*N* = 93)

	Before PSM			After PSM		
	ArtiSential^®^ (*n* = 32)	Straight (*n* = 61)	P value	ArtiSential^®^ (*n* = 32)	Straight (*n* = 20)	P value
**a**						
Operation type			0.062			0.875
Low anterior resection	25 (78.1)	56 (91.8)		25 (78.1)	16 (80.0)	
Ultralow anterior resection	7 (21.9)	5 (8.2)		7 (21.9)	4 (20.0)	
Anastomosis			*0.032			0.378
Stapling	26 (81.3)	58 (95.1)		26 (81.3)	18 (90.0)	
Hand sewing	6 (18.8)	3 (4.9)		6 (18.8)	2 (10.0)	
Diverting stoma			*0.007			0.337
Yes	22 (68.8)	24 (39.3)		22 (68.8)	11 (55.0)	
No	10 (31.3)	37 (60.7)		10 (31.3)	9 (45.0)	
Conversion			0.466			0.33
Yes	0 (0)	1 (1.6)		0 (0)	1 (5.0)	
No	32 (100)	60 (98.4)		32 (100)	19 (95.0)	
Operative time (min)	147.2 ± 46.7	188.1 ± 57.9	*0.001	147.2 ± 46.7	193.6 ± 76.1	*0.021
EBL (mL)	128.3 ± 115.1	169.7 ± 167.0	0.213	128.3 ± 115.1	213.5 ± 244.0	0.156
Intraoperative transfusion			0.202			0.33
Yes	0 (0)	3 (4.9)		0 (0)	1 (5.0)	
No	32 (100)	58 (95.1)		32 (100)	19 (95.0)	
**b**						
Histology			> 0.999			0.648
WD/MD	30 (93.8)	56 (91.8)		30 (93.8)	18 (90.0)	
PD/Mucinous	2 (6.3)	5 (8.2)		2 (6.3)	2 (10.0)	
AJCC Stages			0.868			0.215
0/I	14 (43.8)	22 (36.1)		14 (43.8)	11 (55.0)	
II	7 (21.9)	13 (21.3)		7 (21.9)	5 (25.0)	
III	9 (28.1)	22 (36.1)		9 (28.1)	4 (20.0)	
IV	2 (6.3)	4 (6.6)		2 (6.3)	0 (0)	
Pathologic T category			0.767			0.683
T0/1	8 (25.0)	14 (23.0)		8 (25.0)	5 (25.0)	
T2	10 (31.3)	14 (23.0)		10 (31.3)	7 (35.0)	
T3	12 (37.5)	27 (44.3)		12 (37.5)	8 (40.0)	
T4	2 (6.3)	6 (9.8)		2 (6.3)	0 (0)	
Pathologic N category			0.507			0.431
N0	21 (65.6)	37 (60.7)		21 (65.6)	16 (80.0)	
N1	10 (31.3)	18 (29.5)		10 (31.3)	3 (15.0)	
N2	1 (3.1)	6 (9.8)		1 (3.1)	1 (5.0)	
Harvested LNs	15.3 ± 4.7	19.3 ± 8.3	*0.004	15.3 ± 4.7	20.0 ± 9.3	*0.048
Harvested LNs			0.756			0.567
≥ 12	29 (90.6)	54 (88.5)		29 (90.6)	17 (85.0)	
< 12	3 (9.4)	7 (11.5)		3 (9.4)	3 (15.0)	
TME quality			0.639			0.325
Complete	31 (96.9)	60 (98.4)		31 (96.9)	20 (100.0)	
Nearly complete	0 (0)	0 (0)		0 (0)	0 (0)	
Incomplete	1 (3.1)	1 (1.6)		1 (3.1)	0 (0)	
CRM (mm)			0.166			0.648
≤ 1	2 (6.3)	10 (16.4)		2 (6.3)	2 (10.0)	
> 1	30 (93.8)	51 (83.6)		30 (93.8)	18 (90.0)	
DRM (mm)			0.412			0.556
≤ 5	5 (15.6)	6 (9.8)		5 (15.6)	2 (10.0)	
> 5	27 (84.4)	55 (53.8)		27 (84.4)	18 (90.0)	

EBL, estimated blood loss; PSM, propensity score matching

*p < 0.05. Values are shown as n (%) or mean ± standard deviation

CRM, circumferential resection margin; DRM, distal resection margin; LN, lymph node; MD, moderately differentiated; PD, poorly differentiated; WD, well-differentiated; PSM, propensity score matching

### Postoperative complications

Postoperative complications and early anastomotic leakage (within 30 days) did not differ significantly between the articulating and straight groups (3.1% vs. 1.6%, respectively; *p* = 1.000). The mean length of hospital stay after surgery was 10.8 ± 10.6 days (range, 6–64 days) in the articulating group and 10.3 ± 6.6 days (range, 7–46 days) in the straight group; this difference was not statistically significant (Table 3).

**Table 3:** Postoperative complications (*N* = 93)

	ArtiSential^®^ (*n* = 32)	Straight (*n* = 61)	*P* value
^†^Early complications	13 (40.6)	22 (36.1)	0.822
Wound complications	5 (15.6)	8 (13.1)	0.760
Intraperitoneal infection	3 (9.4)	4 (6.6)	0.689
Anastomotic leakage	1 (3.1)	1 (1.6)	1.000
Urinary complications	3 (9.4)	4 (6.6)	0.689
Postoperative ileus	6 (18.8)	13 (21.3)	1.000
Cardiovascular complications	0 (0)	2 (3.3)	0.544
^§^Clavien-Dindo classifications			0.540
0	25 (78.1)	43 (70.5)	
I/II	3 (9.4)	11 (18.0)	
III/IV	4 (12.5)	7 (11.5)	
Length of hospital stay (days)	10.8 ± 10.6	10.3 ± 6.6	0.752

^†^postoperative days ≤ 30; ^§^early complication

Values are shown as n (%) or mean ± standard deviation

### Long-term survival outcomes

**Fig. 3:**
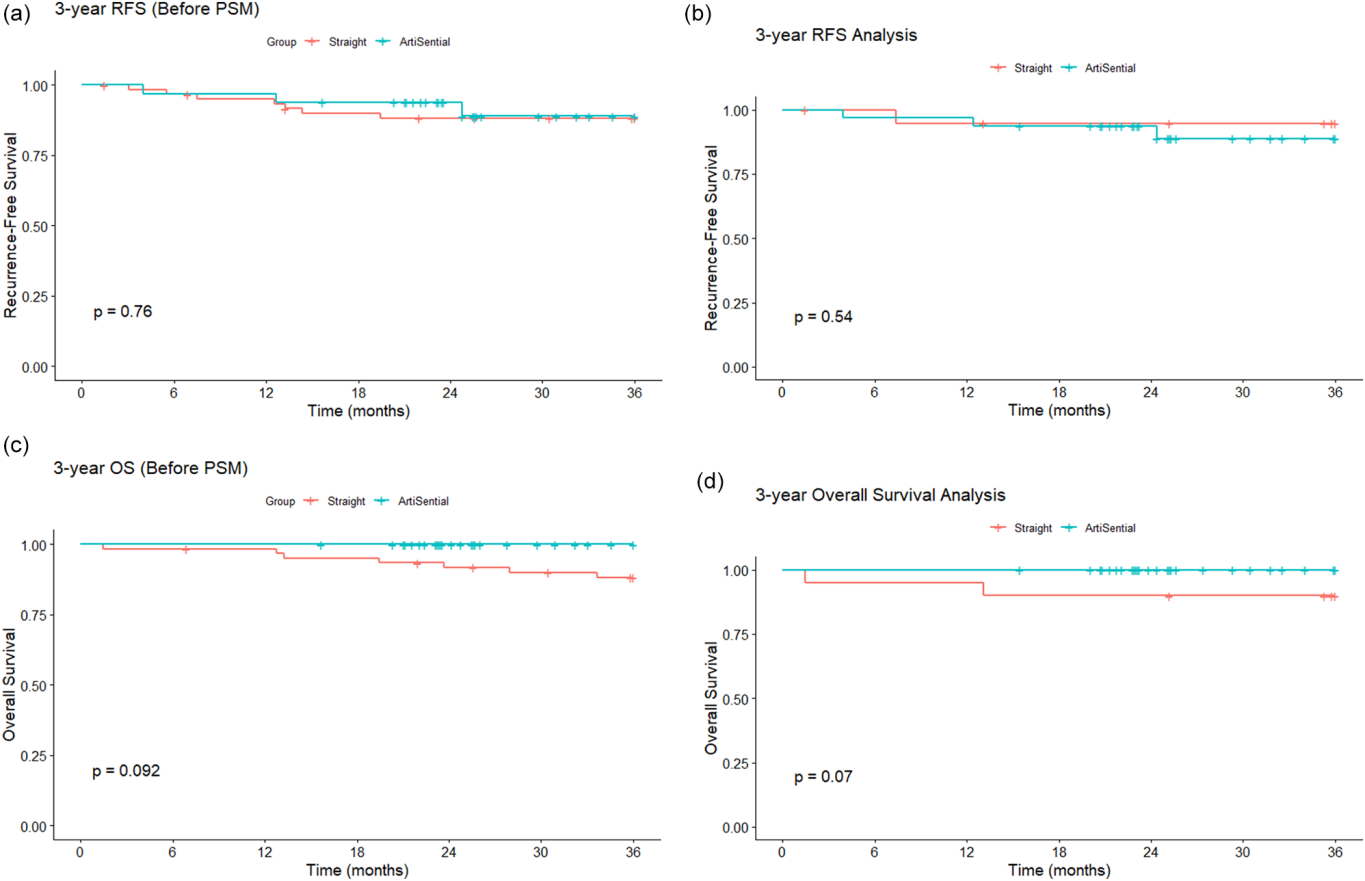
Survival outcomes. (**A**) 3-year Recurrence-free survival (before PSM) (**B**) 3-year Recurrence-free survival (after PSM) (**C**) 3-year Overall survival (before PSM) (**D**) 3-year Overall survival (after PSM)

The median follow-up duration was 45 months (range, 1–140 months). It remained shorter in the articulating group than in the straight group (29 [range, 23–50] vs. 60 [range, 1-140] months, respectively). Recurrence developed in one (3.1%) and three (4.9%) patients in the articulating and straight groups (*p* = 0.202), respectively; all were distal recurrences. The median time to recurrence was 7 months (range, 5–19 months). The 3-year recurrence‐free survival (RFS) rate was 90.6% in the articulating group and 88.5% in the straight group (*p* = 0.760), and the 3‐year overall survival (OS) rate was 100% in the articulating group and 85.2% in the straight group (*p* = 0.092). After PSM, 3-year RFS was 88.8% vs. 94.7% (*p* = 0.54) for articulating and straight group, and 3-year OS was 100% vs. 90% (*p* = 0.07) for articulating and straight group, respectively. Furthermore, the RFS and OS rates did not differ significantly between groups (Fig. 3).

## Discussion

This study demonstrated that operative time could be reduced by the use of a flexible articulated instrument (ArtiSential^®^) in laparoscopic surgery for rectal cancer. Postoperative complications, including anastomotic leakage and deep organ infection, did not differ between the use and straight groups. Oncologic safety could be verified if the operative histopathologic characteristics, including TME quality, were acceptable, and survival outcomes did not differ between groups.

Since Dr. Heald introduced TME in 1988 [21], it has advanced remarkably, and survival outcomes have improved [22]. Minimally invasive surgery is a recent evolution with the short-term benefits of early recovery and comparable long-term survival outcomes. Laparoscopic surgery for rectal cancer was first deemed safe and feasible in the COREAN trial, in which its use did not increase the oncologic risk of circumferential resection margin positivity or the macroscopic quality of TME specimens. During long-term follow-up, the survival outcomes were similar to those of open surgery. The conversion rate was 1.2% in the articulating group versus 1.6% in the straight group.

However, these excellent results have been criticized because the outcomes of the COREAN trial were achieved by highly skilled and well-trained surgeons. The Colorectal Cancer Laparoscopic or Open Resection II (COLOR II) trial results also suggested that laparoscopic surgery provided similar oncologic safety, tumor resection margins, and completeness of resection compared to those of open surgery; moreover, recovery was early after laparoscopic surgery in selected patients treated by skilled surgeons despite the 17% conversion rate. The Laparoscopic-Assisted Resection or Open Resection in Treating Patients with Rectal Cancer (ACOSOG-Z6051) study and the Australasian Laparoscopic Cancer of the Rectum Trial (ALaCaRT) failed to meet the criteria for non-inferiority of pathological outcomes after successful resection.

Due to the narrow and deep nature of the pelvis, laparoscopic TME can be challenging. The complex structure of the pelvic bones renders certain resection sites unreachable using straight laparoscopic instruments. This can increase the risk of intraoperative bleeding and perforation [23]. Preoperative assessments or calculations of the pelvic area using computed tomography or magnetic resonance imaging have been used to predict the difficulties of proposed surgical procedures; thus, a narrow pelvis can be a strong independent factor associated with prolonged operative times [24–26]. Moreover, postoperative specimen quality is poor and local failure rates are high in patients with a narrow pelvis [27, 28].

The newly developed flexible articulated laparoscopic device, the ArtiSential^®^, was initially used in gastrectomy and thoracic surgery [29–31] but has spread to many different surgical fields [32–36]. The device was introduced as an ergonomic surgical instrument with a multi-joint structure, providing a full 360° range of freedom in movement. The double-jointed end effector allows the user to control the instrument in all directions, such as at 90°, which is unachievable with conventional products. The end effector moves synchronously with the user’s hand, wrist, and finger movements, thereby providing intuitively controlled articulation. These end-effectors have both vertical and horizontal joint structures that are synchronized with the delicate movements of the user’s hands and allow improved access to narrow surgical sites [20].

A flexible articulated device can also be used in laparoscopic colorectal surgery, and its potential benefits are especially useful in obese patients with a narrow pelvis [37]. As straight instruments cannot reach the exact mesorectal plane between the rectum and pelvic floor muscle within the deep pelvis, trimming the far distal rectum on both sides to ensure a safe rectal transection with a linear stapler can be difficult and time-consuming. Conflict between the camera and straight instruments can make surgery difficult to perform safely, which can be a reason for conversion to open surgery. The articulating instruments allow smoother access to areas of the pelvic floor that are challenging to reach with straight instruments, particularly the lower lateral regions. This enhanced maneuverability reduces operative time by enabling more efficient and precise dissection in these confined spaces. The use of a flexible articulated instrument can be helpful in these situations; in fact, we reduced the operative time by approximately 40 min without conversion in this study. Also, when a new device is introduced, overcoming the learning curve is essential. However, a number of dry lab practices for this articulating instrument before real use in the operating room would be sufficient for experts in laparoscopic surgery. In fact, our data shows that there were no substantial differences between the initial cases and the later ones. Although patients in whom flexible articulated instruments were used were more likely to require nCRT, their postoperative complications and hospital stays were not significantly different from those in whom the instruments were not used. Oncological safety can be demonstrated by operative histopathology; however, long-term survival outcomes should be monitored.

Robotic surgery has been attempted to overcome the limitations of motion, lack of joint action, and steep learning curve associated with laparoscopic surgery [38]. However, the RObotic vs. LAparoscopic Resection for Rectal cancer (ROLARR) trial could not verify the advantages of robotic versus laparoscopic rectal cancer surgery with similar conversion rates [18]. Some other disadvantages might be relevant, such as high cost, bulky platform, lack of tactile sense, limited instrumentation, and longer robotic surgery duration. The recent Comparison Of Laparoscopic versus Robot-Assisted surgery for Rectal cancer (COLRAR) trial was unable to confirm any improvement in TME quality in robotic versus laparoscopic surgery [39]. To manage the complexity of lower rectal cancer surgery in robotic surgery, transanal TME has recently gained traction, suggesting the feasibility of conversion to open surgery and operative times comparable to those of laparoscopic TME [40]. However, a steep learning curve may still be involved, and the oncological superiority of this technique requires evidence in ongoing trials [17].

Recently, reduced-port and single-port surgeries have been gaining attention for their minimal invasiveness and improved cosmetic outcomes. The ArtiSential^®^ instrument could have potential applications in these non-conventional laparoscopic procedures. Studies, particularly those from Asian researchers, have highlighted positive outcomes for single-port and reduced-port techniques in colorectal surgery, including reduced postoperative pain and higher patient satisfaction [41, 42]. With its multi-jointed design and enhanced dexterity, The ArtiSential^®^ device addresses key technical challenges in reduced-port surgery, such as limited triangulation and conflicts between instruments. Its ability to mimic the natural motion of the human wrist and perform precise movements in confined spaces can help maintain surgical accuracy and efficiency. Furthermore, the ergonomic benefits of the flexible instrument may reduce physical strain on surgeons, a common concern in single- and reduced-port laparoscopic techniques. The demonstrated benefits of reduced operative time and preserved surgical quality suggest that flexible articulated instruments like ArtiSential^®^ could further improve outcomes in reduced-port colorectal surgeries, potentially making these approaches more practical and widely adoptable.

This study has several limitations due to its retrospective design and lack of randomization.

First, the sample size is relatively small, with only 93 patients with an imbalance between the two groups (61 in the straight group and 32 in the articulating group).

Although the primary aim of this study was to compare short- and long-term outcomes between the articulating and straight-instrument groups, we acknowledge that small sample size and baseline imbalances (e.g., TNM staging, neoadjuvant therapy rates, prophylactic stoma formation) limit the generalizability of our findings. Additionally, for long-term survival analysis, we relied on a Kaplan–Meier approach after PSM rather than performing a Cox regression on the entire cohort, as our limited sample size and the notable group size imbalance would likely compromise the stability of a multivariable Cox model. We acknowledge that this method may be statistically less robust, and future larger-scale studies could use more powerful approaches, such as Cox regression, to further validate our findings.

Second, the difference in study periods for the two groups (10 years vs. 3–4 years) may have introduced selection bias and reflects potential improvements in surgical techniques and operator experience over time, making direct comparisons challenging. Significant advancements made during the study period in perioperative management, surgical technology, and visualization systems may have influenced surgical outcomes. Additionally, the experience and performance of the operating surgeon likely improved over the ten-year period, potentially impacting operative times and procedural efficiency. This evolution in practice is a potential source of bias and should be considered when interpreting the observed reduction in operative time with the use of flexible articulated instruments. However, there have been no significant changes in the laparoscopic systems or instruments used, nor any other factors that could have substantially impacted operative time. As shown in the graph, after the initial 30 cases to overcome the learning curve, operative times remained stable until 2020, with only minor variations based on individual cases. From 2020 onward, however, we observed a significant reduction in operative time with the introduction of the ArtiSential^®^ instrument. This reduction, without any other influencing factors, highlights the benefit of using an articulated instrument and represents a key strength of our study.

Third, the follow-up period for the experimental group was shorter due to the recent adoption of the ArtiSential^®^ device (2020–2022). This shorter follow-up limits the ability to draw definitive conclusions about the long-term oncological outcomes for patients treated with flexible articulated instruments.

Regarding our CRM positivity rate (16.4%) in the straight-instrument group, large-scale data from other regions indicate comparable ranges (approximately 8–17%) across various study populations [43, 44]. Moreover, because our institution serves as a quaternary referral center for advanced and complex rectal cancer cases, a higher proportion of high-risk disease may be represented in our cohort. Nonetheless, our relatively low local recurrence rate suggests that elevated CRM positivity does not always necessarily imply poorer oncological outcomes, particularly in the setting of proper neoadjuvant therapy and precise surgical techniques.

Currently, a flexible articulated instrument is used in almost all rectal cancer surgeries, and more advanced cases may be included as the surgeon’s experience and skills increased. Considering that the flexible articulated instrument was used in more difficult cases, this only emphasizes its positive attributes. It would also have been relevant to evaluate and compare the specific duration of TME; however, due to the retrospective nature of this review and lack of detailed records, we could analyze only the total operative time. Pelvic dissection is the most difficult procedure in laparoscopic rectal cancer surgery, and the operative time can serve as a surrogate marker of surgical skill. Some recent work has also suggested that articulated or robotic instruments may provide particular advantages in male patients with mid-low rectal cancer who often present with a narrow pelvis, potentially improving TME completeness and reducing local recurrence. However, whether these benefits extend equally to patients with broader pelvic anatomy, including many female patients, remains an open question. In our present study, the flexible articulated device was used successfully across varying pelvic anatomies, reducing operative times and maintaining acceptable oncological outcomes. Further prospective randomized controlled trials with larger sample sizes and balanced follow-up periods are needed to elucidate the long-term benefits of this flexible articulated instrument in laparoscopic rectal cancer surgery.

## Conclusions

This study demonstrated that flexible articulated instruments may reduce operative time during laparoscopic rectal cancer surgery without compromising oncologic outcomes, suggesting the enhanced efficacy of the device in dissecting challenging surgical planes during TME in laparoscopic rectal cancer surgery. However, because this was a single-institution study led by an experienced surgeon, further multicenter or prospective trials are necessary to confirm these results, verify the proper patient selection variables, and determine the specific technical features responsible for the observed reduction in operative time.

## Electronic supplementary material

Below is the link to the electronic supplementary material.

Supplementary Material 1: Supplement 1. Video clip of laparoscopic low anterior resection using the ArtiSential^®^.

Supplementary Material 2

## Data Availability

The datasets used and/or analyzed during the current study are available from the corresponding author on reasonable request.

## Abbreviations

AV: Anal Verge
BMI: Body Mass Index
CEA: Carcinoembryonic Antigen
CRM: Circumferential Resection Margin
DRM: Distal Resection Margin
EBL: Estimated Blood Loss
LN: Lymph Node
MD: Moderately Differentiated
nCRT: Neoadjuvant Chemoradiotherapy
OS: Overall Survival
PD: Poorly Differentiated
RFS: Recurrence-Free Survival
TME: Total Mesorectal Excision
WD: Well-Differentiated

## Declarations

### Meeting presentation

The results of this study were presented at the 56th Annual Meeting of the Korean Society of Coloproctology held in Gyeongju, Korea, from March 31 to April 2, 2023.
